# Editorial: Co-occurrence of numerical and structural aberration—small supernumerary marker chromosomes and B-chromosomes

**DOI:** 10.3389/fgene.2024.1408674

**Published:** 2024-04-17

**Authors:** Thomas Liehr

**Affiliations:** Jena University Hospital, Friedrich Schiller University, Institute of Human Genetics, Jena, Germany

**Keywords:** small supernumerary marker chromosomes (sSMCs), B-chromosomes, gain of copy numbers, evolution, molecular cytogenetics

This Editorial is for the Research Topic dedicated to small supernumerary marker chromosomes (sSMCs) and B-chromosomes (Bs). Both, sSMCs and Bs share several features in common and it is yet unclear if among human sSMCs there are Bs included. Most strikingly, Bs and sSMCs are at the same time numerical and structural aberrations and may or may not lead to phenotypic changes in the carrier (Liehr, 2023; Liehr, 2024).

sSMCs in humans are deviations from the standard karyotype of 46,XX or 46,XY. sSMCs can originate from each chromosome, contain heterochromatic and/or euchromatic material, and the latter can be derived from one or several chromosomes. sSMCs can have no, minor or major clinical consequences for the carrier (Liehr, 2023; Liehr, 2024). Still, the best way to identify them is banding cytogenetics followed by combined molecular cytogenetics and chromosomal microarray (CMA) studies (Liehr, 2023). Besides, optical genomic mapping and modern high throughput sequencing approaches also are applied for sSMC studies (Weber et al., 2022). Even though progress in genotype-phenotype correlation of sSMCs has been achieved during the last ∼10 years, still many questions are open. Research is also necessary concerning their formation, mitotic stability, and meiotic behavior, as highlighted elsewhere (Liehr, 2023).

In this Research Topic reports were invited on yet unpublished sSMC cases together with their clinical characteristics, insights on how sSMCs are performed nowadays with classical and modern approaches, how a centromere near CNV (gain of copy numbers) picked up in CMA is leading to sSMC-characterization. Single and multiple case reports, as well as studies on cell lines and any kind of study suited to enlighten the centromere near regions in terms of their dosage-dependent gene content. Finally, as sSMCs share several characteristics with Bs, which can be observed in many animal and plant species, besides sSMC- also Bs related research with a potential impact on sSMC field would have been welcome for this Research Topic. As no such papers were obtained interested readers are referred to a recent review in that Research Topic (Oliveira et al., 2024).

However, the following important Research Topic concerning sSMCs have been covered here by six high quality contribution from Germany (Liehr et al.), Mexico (Navarette-Menses et al.), Morocco (Ouboukss et al.), Russia (Karamysheva et al.), Spain (Rodríguez) and Serbia (Joksic et al.).

The difficulties to characterize sSMCs in general were highlighted excellently by Laura Rodriguez (Rodríguez) by reviewing such problems from (Liehr, 2024), including heterochromatic sSMCs with or without uniparental disomy (UPD), euchromatic sSMCs, which, if ring shaped always need to be checked for McClintock mechanism, or centromere misdivision of a chromosome in connection with sSMCs.

Ivana Joksic et al. report 12 sSMC cases from Serbia characterized by different approaches and derived from variant chromosomes. They also highlight the problem, that a normal diagnostic lab still nowadays may not be able to solve each sSMC case completely.

Thomas Liehr et al. report new cases with sSMCs derived from chromosome 11 and provide a comprehensive review of the literature of this rare sSMC-group. It is shown, that pericentric triplo-sensitive and insensitive regions can be a key for understanding clinical effects in patients with this kind of rare numerical and structural chromosomal aberration.

Fatima Ouboukss et al. report a genotype phenotype correlation attempt in a complex sSMC derived from chromosome 14, with parts of chromosome 8. As in most complex sSMCs also this actual case was derived from a parental (here maternal) balanced translocation and a meiotic 3:1 segregation.

Karamysheva et al. deal with an unusual case of Pallister-Killian syndrome, highlighting the fact that the cytogenetic variance in sSMC-syndromes may be much higher than in other chromosomic syndromes.

Finally, Navarette-Menses et al. bring back to our attention that pigmentary skin mosaicism may be attributed in a good part of the corresponding cases to sSMCs, derived from different chromosomal origins. The latter is a good example that a chromosomal imbalance may lead to (similar) phenotypic consequences not because of “the one specific gene” to be identified by next-generation sequencing, but as a consequence of a gain or loss of copy numbers in the range of megabasepairs. Such events seem to lead to a general, yet not understood cellular answer, rescuing cells with massive loss of gain of copy numbers. Accordingly, a search for ‘the disease causing gene’ in Patau syndrome, Down syndrome, Edwards syndrome, ring chromosome syndromes (Li and Liehr, 2024) or specific sSMC-related syndromes will not be successful, as long as observatons like these reported here are not considered, or more precisely ignored by main stream of human (molecular) genetics.

Overall, this Research Topic brought together a small collection of important reports, highlighting the great variance in sSMCs in general.
